# Full Design Automation of Multi-State RNA Devices to Program Gene Expression Using Energy-Based Optimization

**DOI:** 10.1371/journal.pcbi.1003172

**Published:** 2013-08-01

**Authors:** Guillermo Rodrigo, Thomas E. Landrain, Eszter Majer, José-Antonio Daròs, Alfonso Jaramillo

**Affiliations:** 1 Institute of Systems and Synthetic Biology, CNRS UPS 3509 – Université d'Évry Val d'Essonne – Genopole, Évry, France; 2 Instituto de Biología Molecular y Cellular de Plantas, CSIC – Universidad Politécnica de Valencia, Valencia, Spain; Lawrence Berkeley National Laboratory, United States of America

## Abstract

Small RNAs (sRNAs) can operate as regulatory agents to control protein expression by interaction with the 5′ untranslated region of the mRNA. We have developed a physicochemical framework, relying on base pair interaction energies, to design multi-state sRNA devices by solving an optimization problem with an objective function accounting for the stability of the transition and final intermolecular states. Contrary to the analysis of the reaction kinetics of an ensemble of sRNAs, we solve the inverse problem of finding sequences satisfying targeted reactions. We show here that our objective function correlates well with measured riboregulatory activity of a set of mutants. This has enabled the application of the methodology for an extended design of RNA devices with specified behavior, assuming different molecular interaction models based on Watson-Crick interaction. We designed several YES, NOT, AND, and OR logic gates, including the design of combinatorial riboregulators. In sum, our *de novo* approach provides a new paradigm in synthetic biology to design molecular interaction mechanisms facilitating future high-throughput functional sRNA design.

## Author Summary

Is our current knowledge of *in vivo* RNA-RNA interactions and thermodynamics enough to perform the unsupervised computational design of fully synthetic sequences encoding functional RNAs in living cells? Recent work gave a positive answer for the challenging problem of designing activating riboregulators. This was done by integrating theory and computation to develop a physicochemical framework for the design of regulatory RNA systems, using Watson-Crick interactions and optimization algorithms. Still, the objective function was not directly validated, preventing using with confidence the methodology for other systems. We here validate experimentally an objective function relying on free energies of RNA complex activation and formation, which allows extending the framework to produce logic devices that can be implemented to program gene expression. We demonstrate that it is possible to design increasingly sophisticated and modular functions, pointing our results out that energy-based optimization methods can perform the large combinatorial search required for RNA design.

## Introduction

Small non-coding RNA (sRNA) has raised a big interest because of the predictability and modularity of its binding with a large variety of molecules and macromolecules [1]. Given this functional potential, the use of sRNAs to control protein expression has triggered a new way to engineer integrated regulatory networks [2]. Although rational techniques have been successfully applied to redesign natural systems [3], [4], engineer synthetic ones [2], [5]–[7] and assemble modular structures [8]–[10], *de novo* sequence design still remains difficult because of the size and complexity of multi-state systems. To overcome this, we propose an evolutionary computation design strategy [11], where all design specifications are automatically assembled to yield an optimal solution.

In this work, we demonstrate a full design automation of RNA sequences that implement diverse riboregulatory mechanisms, able to produce several sRNA-based logic gates that are functional in living cells. We generalize our previous work [11] on the design of riboregulators for activating protein expression, which could be considered as YES gates, to derive objective functions to design riboregulators implementing several logic gates. Furthermore, we experimentally validate our objective function by considering mutants of natural and synthetic riboregulators [11], [4], and this allows assessing the generality of the methodology.

**Figure 1:**
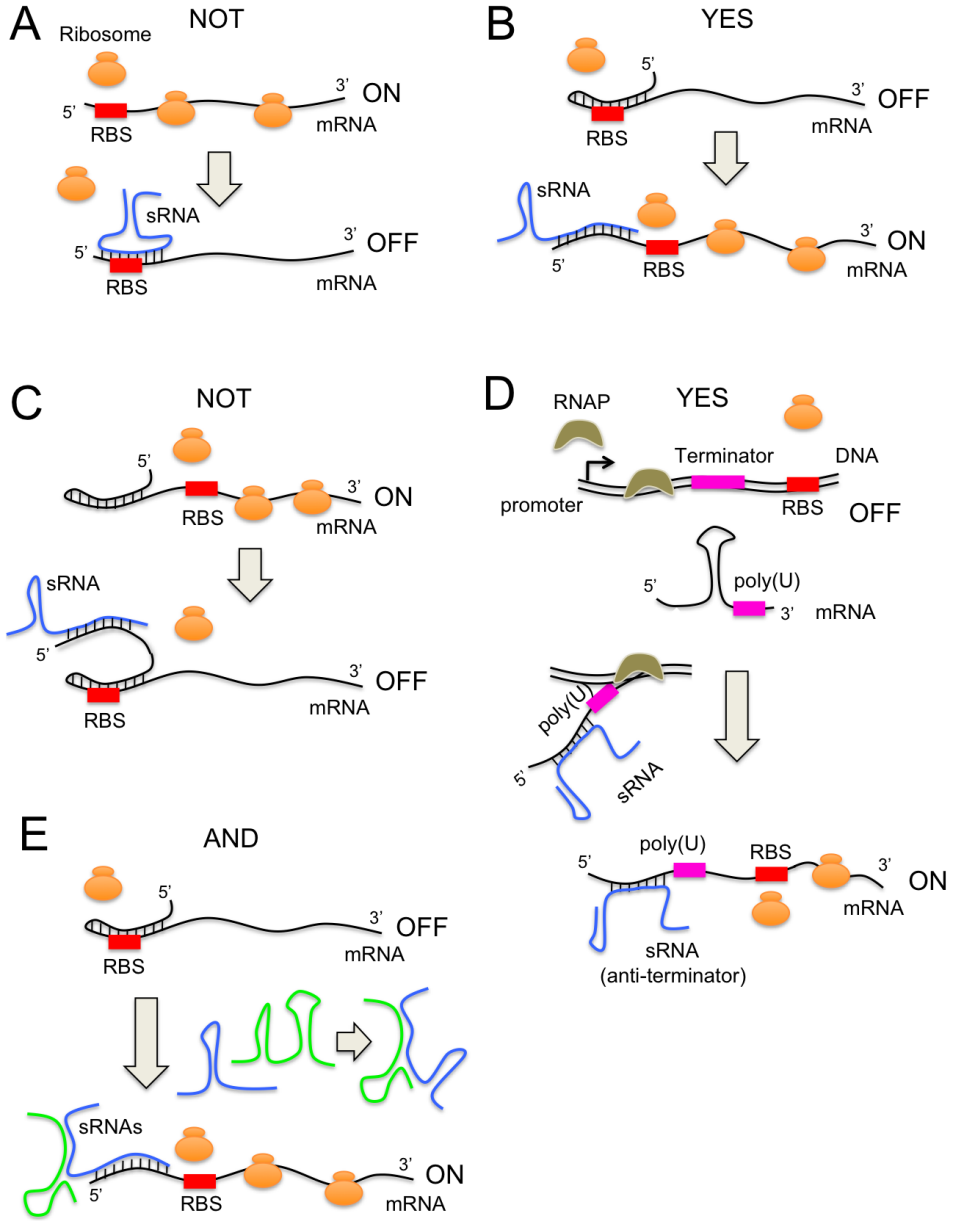
Schemes of different sRNA-based mechanisms to control protein expression. Riboregulation is based on conformational changes in the secondary structures of RNA molecules that allow controlling protein expression. The annealing mechanism between two sRNAs starts by the nucleotides in the seed to form an intermediate complex and then follows to reach the structure of minimal energy. (A) Scheme of a NOT logic gate, which consists in an sRNA able to bind to the RBS sequence to block translation. (B) Scheme of a YES logic gate, where the sRNA is designed to release the RBS that is *cis*-repressed. (C) Scheme of a further NOT logic gate, where the sRNA is able to induce *cis*-repression (exploiting the mechanism shown in B). (D) Scheme of a further YES logic gate, where the sRNA interacts with a transcription terminator placed upstream of the RBS, allowing or preventing the formation of the mRNA. (E) Scheme of an AND logic gate, where two sRNAs are designed to interact among them and form a complex that can release the RBS.

By generalizing the positive riboregulation paradigm, where an sRNA interacts through Watson-Crick pairing with a target mRNA to trigger a conformational change enabling ribosome docking, we can extend the methodology to design arbitrary logic gates, accounting for new regulatory mechanisms, such as anti-termination, and implementing constrained design strategies (Fig. 1). For that, we exploit antisense and allosteric RNA [12], [13], two conserved mechanisms based on precise secondary structures, and whose major role has been reported over the last years in bacteria [14], but also in humans [15] and plants [16]. Our method starts from random sequences to proceed with successive rounds of a mutation operator, followed by selection using an objective function that accounts for the free energies of all possible reactions and the secondary structures of all species. Previous work on full design automation of nucleic acids was focused on *in vitro* annealing of small DNAs [17]–[20], hammerhead ribozymes [21], or ribosome binding sites (RBSs) [22].

In the following, we will start by formulating the RNA design problem as an inverse problem to program gene expression. This is based on an optimization method that minimizes an *ab initio* objective function, which contrasts with other approaches [4]. We will evaluate such an objective function by engineering and characterizing our own mutant library of synthetic riboregulators activating gene expression. Afterwards, we will show and exemplify how to design sRNA-based logic gates, including complex gates involving synergistic interactions of different sRNAs as inputs. Finally, we will discuss the results stressing the limitations of our methodology.

## Results

### Formulation of an inverse problem

Riboregulation is based on conformational changes, after interaction, in the structures of RNA molecules, which allow controlling protein expression. To design such regulatory RNAs, we optimize the potential energy curve defined in the transition state theory [23], minimizing the free energies of the transition and hybridization states. We assume that the individual folding state is formed before intermolecular RNA-RNA interaction, because its time scale is of milliseconds whereas hybridization takes seconds or even minutes [24], [25]. The interaction mechanism is guided by means of the seed region (nucleation site; the first nucleotides that get paired) to form an intermediate complex at the transition state [3], [11]. Then, both RNAs are destabilized to form a complex with a new structure and minimal energy.

Here, we consider the structures of all individual species as design specifications. To address the computational design, we firstly have to find sequences folding into predefined structures and, second, find sequences able to interact specifically among them to form complexes displaying the correct behavior. The structural constraints are exploited to considerably reduce the combinatorial space and accelerate the design of nucleic acid sequences. Our computational procedure optimizes at the same time all RNA sequences of the circuit. During the optimization, we do not impose constraints in nucleotide sequence, such as stems with high GC-content or loops with YUNR motifs, which have been found in natural systems [12]. Importantly, our designs are just based on basic physicochemical principles and not on additional fitting, allowing the solution of the full design problem.

**Figure 2:**
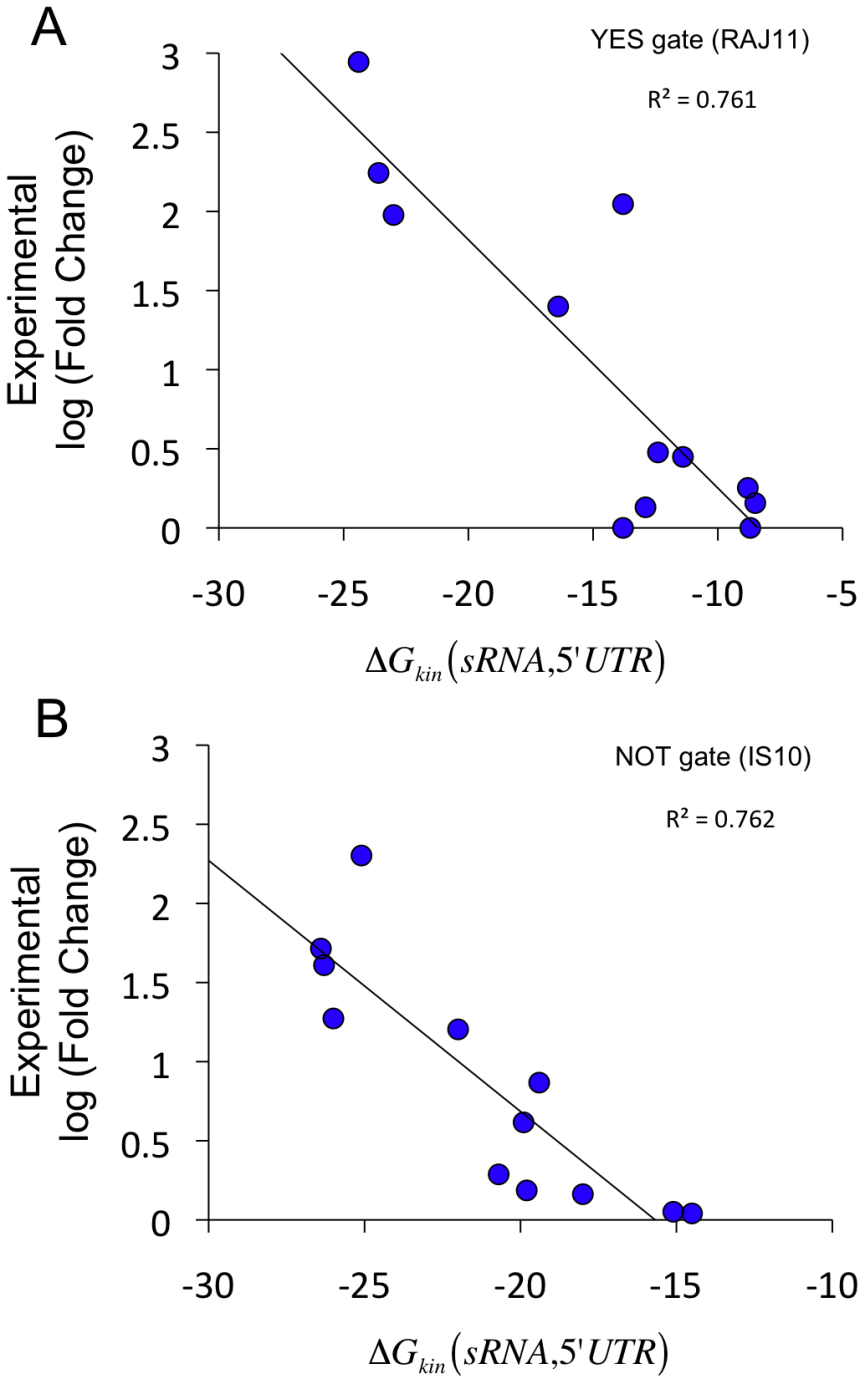
Experimental validation of the objective function. (A) Representation of the log of the experimental activation folds for a set of RNA devices constructed in this work (mutational variants of the RAJ11 system [11]) versus Δ*G*_kin_ (Eq. 13). This system implements a YES logic gate, which was designed with the algorithm presented here (see also Table S4). (B) Representation of the log of the experimental repression folds recently reported for a set of mutational variants of the IS10 antisense RNA system [4] versus Δ*G*_kin_. This system implements a NOT logic gate, and it serves to test the predictability of the method against independent experimental data (see also Table S2). Here, we do not consider Δ*G*_str_ as we are only analyzing the interaction ability. The lines correspond to linear regressions, and the coefficients *R* ^2^ are shown, assuming a model where the fold change scales exponentially with the free energy.

But, is the proposed objective function predictive enough to allow the designability of multi-state RNA devices? To illustrate this question, we constructed here a library of mutants of one of our previously designed circuits (the device RAJ11 [11], implementing a YES logic gate as shown in Fig. 1B). Then, we represented the experimental values of the measured activation fold against the objective function calculated for those mutants (Fig. 2A). To give further support to our objective function, we evaluated it for a set of mutational variants of the IS10 antisense RNA system [4], implementing a NOT logic gate (Fig. 1A), and then we represented those values against the experimental repression folds reported (Fig. 2B). This natural system constitutes an independent validation. The objective function here (Eq. 13) accounted for the free energy of formation and the length of the seed in the sRNA-mRNA interaction. Fig. 2 shows a good correlation (without any fitting) for our objective function and experimental data, which supports the designability of those devices.

### Design of simple sRNA-based logic gates

**Figure 3:**
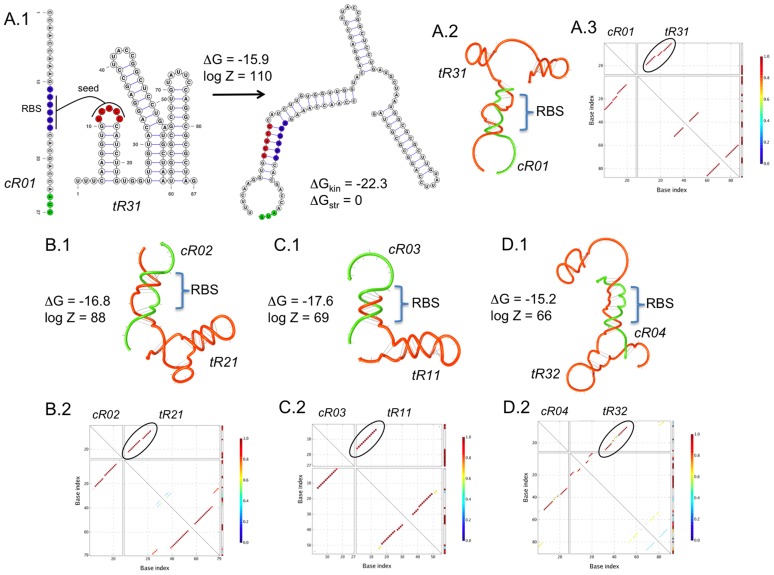
Designs of sRNA-based NOT logic gates. We show four designs (A to D) using different structures for the *trans*-repressing sRNAs (mechanism shown in Fig. 1A). (A.1) Detail of a design, showing the RBS in blue, start codon in green, and seed region in red. The secondary structures of the intramolecular and intermolecular folding states are presented. (A.2, B.1, C.1 and D.1) Helical plot of the complex, where the RBS is blocked. Δ*G*, Δ*G*_kin_ and Δ*G*_str_ are in Kcal/mol. *Z* is the partition function. (A.3, B.2, C.2 and D.2) Base pairing probability matrix, encircling the pairs of intermolecular interaction with high probability. RNA sequences shown in Table S1. Secondary structures imposed for all species shown in Fig. S1.

We first applied our design methodology to obtain sRNA-based repression and activation. Many known riboregulators impart a repressive action on their targets by promoting accelerated degradation through endoribonucleases, which initiate turnover of both RNAs [26]. Instead, we here account for sRNAs that bind specifically to a segment of its target mRNA in order to inhibit translation (NOT logic function) [4]. The most intuitive mechanism consists in blocking the Shine-Dalgarno sequence, which is generally located about eight base pairs upstream of the start codon (AUG), for preventing ribosome docking (Fig. 1A). For instance, in *E. coli* plasmid F, sRNA FinP directly binds to the 5′ untranslated region (UTR) of protein TraJ [12]. We constructed the following objective functions (definitions of Δ*G*_kin_ and Δ*G*_str_ in section Methods) to solve the optimization problem
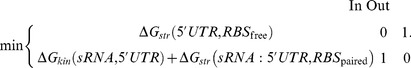
(1)These functions are associated to each entry of the truth Table, and then the solution of this problem will yield NOT logic gates. In Fig. 3, we show several computational designs of this logic device. We applied our methodology with different natural occurring structures involving one, two or three hairpins for the *trans*-repressing sRNAs. In our designs, we used the Shine-Dalgarno sequence AGGAGA.

**Figure 4:**
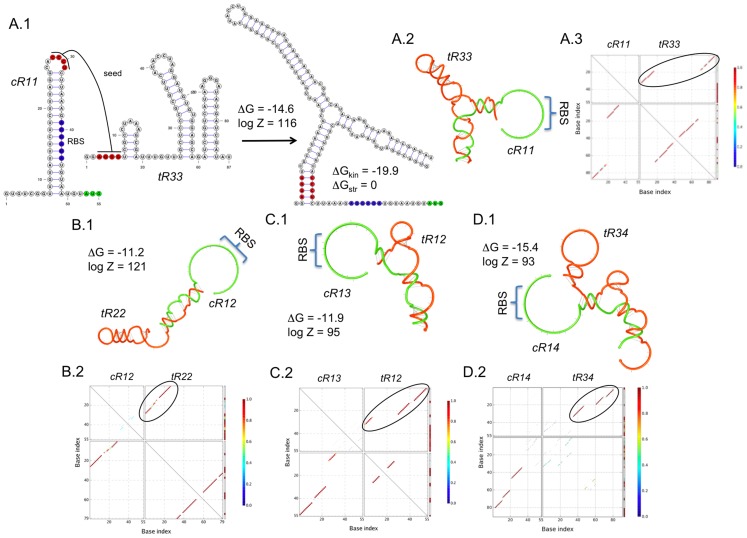
Designs of sRNA-based YES logic gates. We show four designs (A to D) using different structures for the *trans*-activating sRNAs (mechanism shown in Fig. 1B). (A.1) Detail of a design, showing the RBS in blue, start codon in green, and seed region in red. The secondary structures of the intramolecular and intermolecular folding states are presented. (A.2, B.1, C.1 and D.1) Helical plot of the complex, where the RBS is released. Δ*G*, Δ*G*_kin_ and Δ*G*_str_ are in Kcal/mol. *Z* is the partition function. (A.3, B.2, C.2 and D.2) Base pairing probability matrix, encircling the pairs of intermolecular interaction with high probability. RNA sequences shown in Table S1. Secondary structures imposed for all species shown in Fig. S1.

Although the majority of sRNA-mediated regulation in *E. coli* consists in repression, an sRNA can also operate as an activator (YES logic function) [2]. In this case, the sRNA *trans*-activates a *cis*-repressed gene by its 5′ UTR. After interaction, the conformational change in the 5′ UTR releases the Shine-Dalgarno sequence and allows translation (Fig. 1B). For instance, in *E. coli*, sRNA DsrA is responsible of activating the expression of sigma factor RpoS, which modulates the stress response [13]. Hence, we constructed the following objective functions
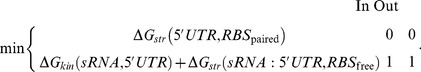
(2)The solution of this problem will produce the intended function specified in the truth Table. This problem is much complex that the previous one because here the two RNA species have structure. In Fig. 4, we show several computational designs of YES logic gates based on conformational changes in the 5′ UTRs of the target genes. We applied our methodology with different structures for the *trans*-activating sRNAs, while maintaining a common structure for the 5′ UTR. We also attempted the computational design of a synthetic RNA able to interact with the RpoS 5′ UTR, and then enhance the translation rate. Fig. S2 shows the sequences and structures obtained.

**Figure 5:**
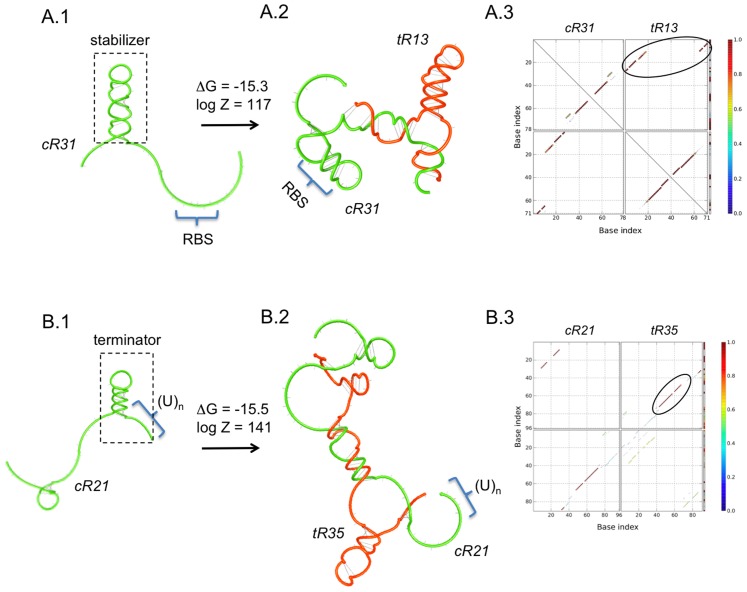
Further designs of sRNA-based NOT and YES logic gates. We show two designs (A and B) using the mechanisms shown in Figs. 1C and 1D. For the NOT gate, helical plots showing (A.1) the RBS exposed, and (A.2) the RBS blocked after sRNA interaction. For the YES gate, helical plots showing (B.1) a transcription terminator, and (B.2) that the hairpin before the poly(U) tail is destabilized after sRNA interaction. Δ*G* is in Kcal/mol. *Z* is the partition function. (A.3 and B.3) Base pairing probability matrix, encircling the pairs of intermolecular interaction with high probability. RNA sequences shown in Table S1. Secondary structures imposed for all species shown in Fig. S1.

In addition, we exploited our methodology to design NOT logic gates based on structured 5′ UTRs. Here, the *trans*-activating sRNA interacts with the 5′ UTR to induce a conformational change that blocks the Shine-Dalgarno sequence (Fig. 1C). The objective functions to solve the corresponding problem read
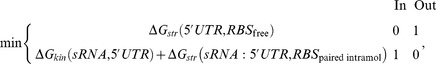
(3)where the difference with Eqs. (1) relies on the imposition that the RBS must be paired at the intramolecular level. Fig. 5A shows a computational design implementing this regulatory mechanism. We also designed riboregulators with activation activity based on a mechanism of anti-termination [27]. This design relies on a *trans*-regulating sRNA able to destabilize the structure of a terminator, which is here the *cis*-regulating element, resulting in a complex that allows the progression of the RNA polymerase (Fig. 1D). This mechanism can also entail kinetic effects [3], where the interaction has to occur before RNA polymerase reads through the terminator. This may impose a narrow time window for operation, which we speculate surmountable provided a given free energy threshold and a high ratio sRNA/mRNA. In this case, the objective functions were
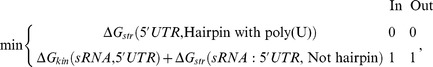
(4)where the 5′ UTR encodes for a terminator that is formed in absence of the sRNA. The solution of this problem will also satisfy the truth Table for YES. Fig. 5B shows a computational design of a YES logic gate based on this mechanism. In the final structure of the complex, the terminator hairpin is destabilized and the poly(U) tail does not have any effect.

### Design of combinatorial sRNA-based logic gates

**Figure 6:**
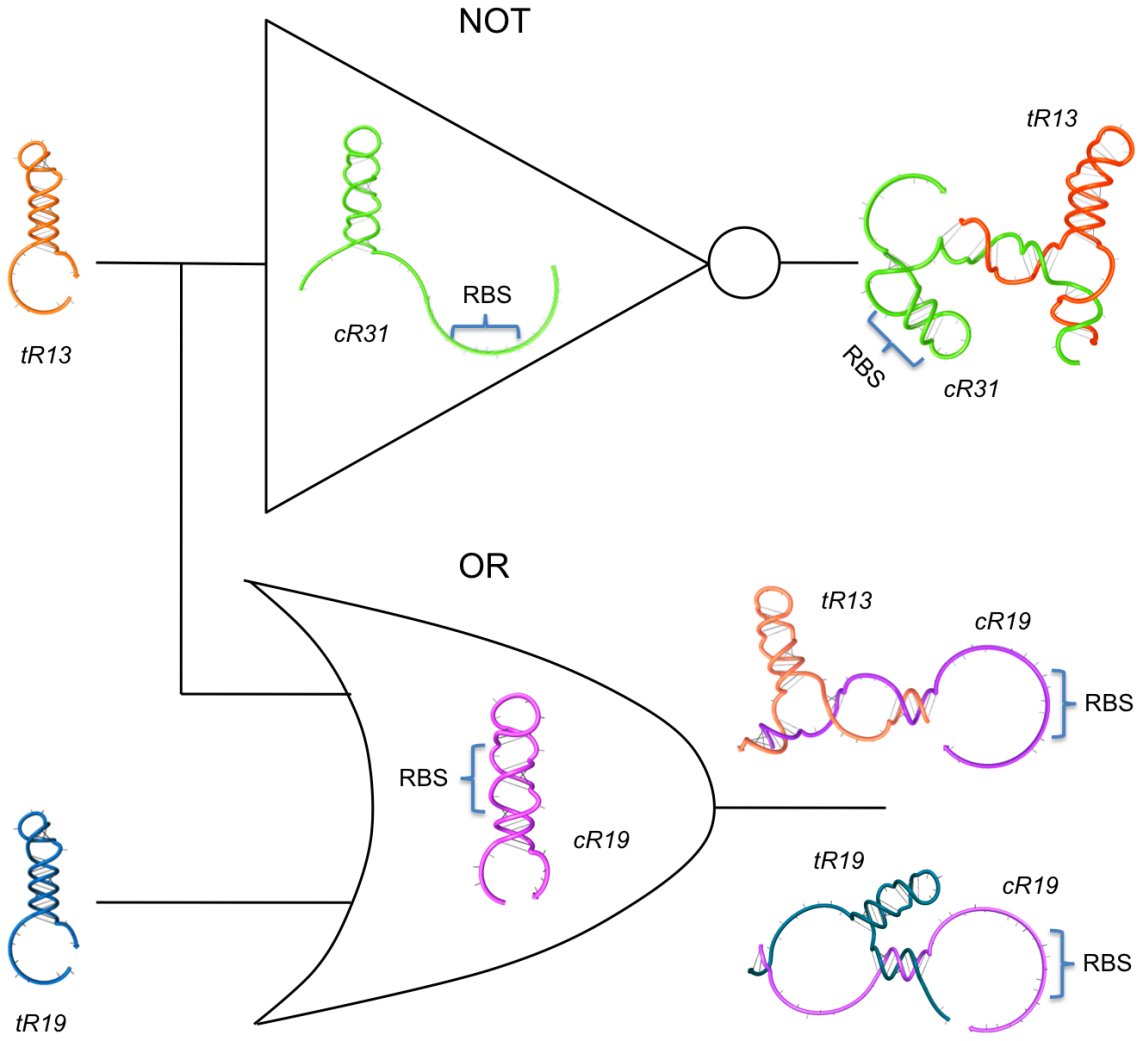
Design of a multi-input, multi-output sRNA-based logic circuit. We show a design of a circuit that assembles different riboregulators. Here, sRNA *tR13* is able to both repress and activate the expression of two different *cis*-repressed genes, by *cR31* and *cR19* respectively, resulting in a coupled YES/NOT logic gate. In addition, sRNA *tR19* is able to activate *cR19*, implementing together with *tR13* an OR logic gate. RNA sequences shown in Table S1. Secondary structures imposed for all species shown in Fig. S1.

We then applied our methodology for the design of higher-order riboregulatory devices. Taking the NOT logic gate shown in Fig. 5A as a reference, we performed the design of a new 5′ UTR for *cis*-repression and that was able to respond to the same riboregulator, in this case working as an activator. The optimization problem read
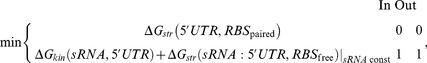
(5)where the difference with Eqs. (2) relies on the imposition that the sRNA sequence is constant. Likewise, the same sRNA will have the ability to both repress and activate protein expression (coupled YES/NOT logic gate). Exploiting further this modularity, we carried out the design of an OR logic gate using the 5′ UTR sequence just designed. We now enforced the design of a new sRNA that had also the ability of releasing the RBS, maintaining constant the 5′ UTR sequence. The optimization problem had then only one instance, given by
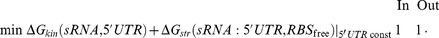
(6)Thus, the resulting system will integrate two sRNAs capable of activating the release of the RBS contained in a single 5′ UTR. Subsequently, we verified there was no interference between the two sRNAs, although this could have also been incorporated into the design process. Fig. 6 shows the integrative circuit (multi-input, multi-output) that we finally obtained with this strategy based on serial design of constrained YES gates.

**Figure 7:**
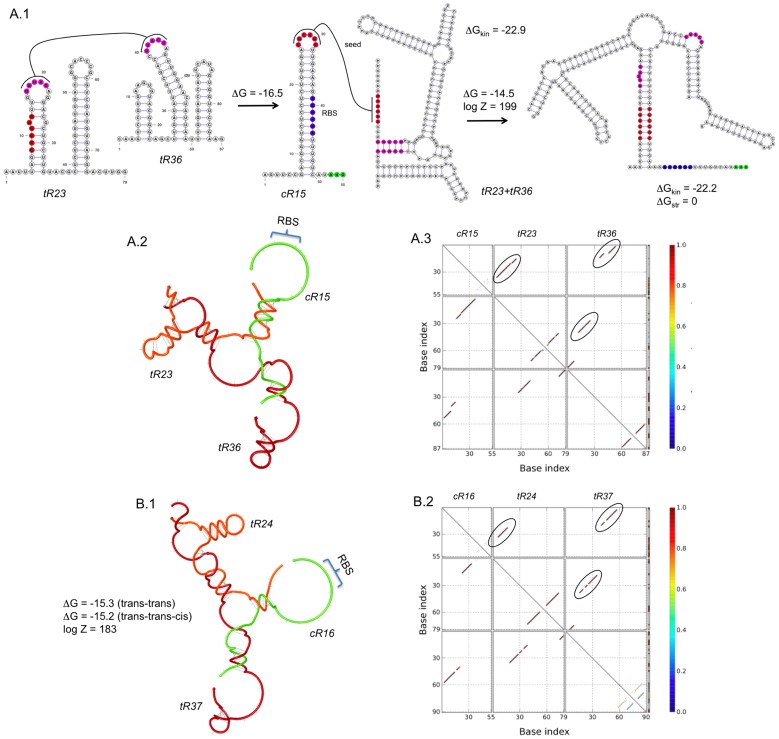
Designs of sRNA-based AND logic gates. We show two designs (A and B) using different structures for the *trans*-activating sRNAs (mechanism shown in Fig. 1E). (A.1) Detail of a design, showing the RBS in blue, start codon in green, and seed regions in red and magenta. The secondary structures of the intramolecular and intermolecular folding states are presented. (A.2 and B.1) Helical plot of the complex, where the RBS is released. Δ*G*, Δ*G*_kin_ and Δ*G*_str_ are in Kcal/mol. *Z* is the partition function. (A.3 and B.2) Base pairing probability matrix, encircling the pairs of intermolecular interactions with high probability. RNA sequences shown in Table S1. Secondary structures imposed for all species shown in Fig. S1.

Motivated by the previous results, we carried out the design of cooperative riboregulations. The regulatory function of multiple-sRNA complexes has not been reported in prokaryotes (all natural systems for riboregulation involve two RNA species, at most interacting with proteins such as RNA chaperones or endoribonucleases [28]), which further encourages the exploration by means of computational methods. To illustrate the power of our approach, we focused on the design of synergistic activation (AND logic function), where two *trans*-regulating sRNAs first interact among them to form a complex that will then activate translation (Fig. 1E). To solve the optimization problem, we constructed the following objective functions
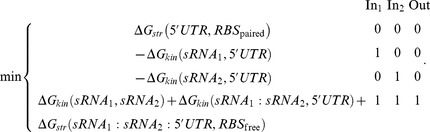
(7)As in the previous cases, these functions are associated to each entry of the truth Table, and hence the solution of this problem will yield AND logic gates. In Fig. 7, we show two different designs of this logic, combinatorial device. By themselves, the *trans*-regulating sRNAs cannot release the RBS. However, the dimer they form has a distinct structure that allows interplaying with the 5′ UTR.

## Discussion

In conclusion, we have followed a bottom-up approach to design RNA devices with YES, NOT, AND, and OR logic functions, based on first physical principles. These logic gates implement multi-state sRNA devices for which there was no design method before, and that can be interconnected to create more complex logic programs. Although we could solve intermolecular inverse folding problems [29], it was not possible the systematic design of multiple RNA species implementing arbitrary logic gates. For their design, each entry of the truth Table imposes a structural specification. Here, we accounted for the free energies of all possible reactions (thermodynamic potential) to solve this multi-objective inverse problem by optimization. Because our methodology does not require natural sequences (with the exception of key motifs such as the Shine-Dalgarno sequence), we have solved the full design problem of regulatory RNA for implementing logic programs in living cells.

Our approach has, however, some limitations, which prospect further research in the field. One of them is the use of the secondary structure to model riboregulation. This type of regulation could involve pseudoknot interactions and even non-canonical base pairing, for which three-dimensional models could better capture the interaction features [30]. In addition, our model does not account for RNA chaperons (e.g., Hfq) [31], nor co-factors such as Mg^2+^ or Zn^2+^, nor kinetic binding effects, which might have an impact on the designs. Another restraint of the current method is the enforcement of a given structure for all single species in the circuit (although not for the complex ones), because this constrains the sequence space of possible solutions [11]. By leaving unconstrained those structures, we could perform additions and/or deletions (not only replacements) of nucleotides during the optimization, and we would need to include into the function Δ*G*_str_ a new term for the stability (e.g., based on free energy). Finally, the convergence of the algorithm is highly reduced when evolving systems with multiple species, making necessary to reduce the sequence space by reusing functional modules to obtain more sophisticated systems.

Despite these limitations, we have demonstrated the power of computational design (through heuristic optimization) to overcome the complexity in obtaining fully synthetic riboregulation, exploring the vast combinatorial space of sequences. The proposed objective function was shown predictive enough to allow the designability of multi-state RNA devices, as Δ*G*_kin_ explained differences in experimental repression fold for a set of mutational variants of the IS10 antisense RNA system (Fig. 2) [4]. Moreover, we recently validated experimentally some designs of YES logic gates in bacteria, encouraging further work [11]. Even though, the design problem does not require a perfect prediction, and similar or even lower correlations can be sufficient to tackle this problem, such as in the case of automated RBS design [22]. Of course, more sophisticated objective functions will be developed in the coming years to improve the design of functional RNAs.

The combination of Δ*G*_kin_ and Δ*G*_str_, for every possible conformational state (intra- or intermolecular) of a given genotype, results in an effective free energy that defines a fitness landscape. In case of riboregulation, the total search space can be about 10^40^ sequences [11], and typical optimizations that lead to sufficiently good solutions consist of 10^6^–10^7^ iterations. Indeed, the generalized problem of finding the nucleotide sequences of multi-species ensembles that will fold into specified conformations has an exponentially large number of solutions. It remains however a question how to distinguish several optimized sequences (assuming equal energetic features). For instance, differences in intracellular stability of the species will affect the ratio sRNA/mRNA, and then be key for the regulatory activity. Additionally, the kinetics of RNA folding, binding, and turnover will have significant impact on the performance of designed RNA circuits [3], [10]. All these criteria, either from first principles or from experimental feedback, will be exploited to enhance the design methodology.

Our present methodology is general and could be applied to obtain designs based on further mechanisms. In addition, instead of attempting full designs, it permits reusing complete known sequences (natural or synthetic) to constrain the design of new logic systems. This capacity enables the creation of a large variety of combinatorial sRNA systems, increasing sophistication at a reduced computational cost. Moreover, our approach can be used to analyze potential RNA sequences for a given functional circuit as a reverse engineering tool. The designed sRNA-based logic gates can be combined with transcription regulation to generate more complex functions [32], and also be integrated into libraries of models for the computational design of more complex networks involving transcription and post-transcription regulation [33]. Yet, our full design automation approach together with high-throughput screening techniques will propel the construction of modular and orthogonal devices for synthetic biology [34].

## Methods

### Thermodynamic model

We considered riboregulation (RNA-RNA interaction) in terms of thermodynamics [29], [35], [36], assuming that the system reaches an equilibrium state. We first applied an inverse folding strategy over the structures of all individual species. Then, neutral mutations in structure were evaluated with an objective function intended to optimize the intermolecular folding states. To obtain an intermolecular folding satisfying the release or blockage of the RBS, in principle, we needed to maximize the partition function (*Z*) of the whole system. Using the reaction coordinate of the system (*r*), defined as the number of intermolecular Watson-Crick interactions (i.e., *r* = 0 represents individual folding) [11], *Z* can be written as
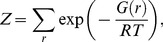
(8)where *G*(*r*) is the effective free energy of the state with reaction coordinate *r* (where *G*(0) represents the free energy of the no-interaction state, with *G = *0 for the unfolded state), *R* the gas constant, and *T* the temperature. Here, we are interested in *G*(*r*) at the reaction coordinates for the transition, *G*(*r_trans_*), and final intermolecular (hybridization) states, *G*(*r_hyb_*), to define our functions Δ*G*, the free energy of formation, and Δ*G* ^‡^, the free energy of activation, by
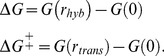
(9)


To compute the free energy and secondary structure of all species (single and complexes) of a system, we used the ViennaRNA [37] and MultiRNAFold [38] (when having more than two RNA species) software. We only considered the minimum free energy state discarding the suboptimal ones. Here, we did not consider pseudoknots. Afterwards, the designed sequences were analyzed with the Nupack software [29], which is able to compute ensemble properties such as *Z*. In this work, we used the Mfold 3.0 RNA energy parameters [39], and always considered *T* = 37°C (which gives *RT* = 0.61 Kcal/mol).

### Deriving a generic objective function for *in vivo* RNA-RNA interactions

In an RNA-RNA interaction between species *A* and *B*, an intermediate complex at the transition state ([*A*:*B*]^‡^) is formed mediated by the seed. Then, a fast reaction inducing a conformational change occurs. Denoting *k_on_* and *k_off_* the forward and reverse constants, respectively, to form [*A*:*B*]^‡^, and *k_hyb_* the hybridization constant to form the final complex (*A:B*), the mass action kinetic model reads
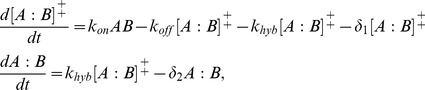
(10)where *δ*_1_ and *δ*_2_ are the degradation constants. Assuming that *k_off_* + *k_hyb_* is much greater than *δ*_1_ (sRNA degradation takes several minutes [13]), we can obtain in steady state [*A*:*B*]^‡^ = *AB*/*K_M_*, where *K_M_ = *(*k_off_* + *k_hyb_*)/*k_on_* is the Michaelis constant. Hence, *A:B* (and also the translation rate) will be in steady state proportional to *k_hyb_*/*K_M_*, assuming there is no saturation.

The constant *k_on_* can be obtained by fitting *in vitro* DNA hybridization data, where only the length of the seed (*α*), irrespective to the sequence, determines the kinetic constant following a Boltzmann factor [25]. Moreover, we can say that the constant *k_hyb_* is determined by Δ*G* (the free energy of formation between *A* + *B* and *A:B*) also with a Boltzmann factor. This allows us to write
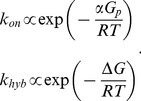
(11)Therefore, the resulting model reads
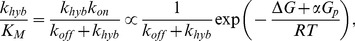
(12)where *G_p_* is a fitted parameter to account for the average energetic contribution of one nucleotide. *G_p_* = −1.28 Kcal/mol [25]. Finally, we proposed Δ*G* + *αG_p_* as the objective function to optimize RNA-RNA interactions. This formulation is in part equivalent to maximize *Z*, because from the Arrhenius equation [23] Δ*G* ^‡^ and *α* should have a linear relationship.

### Optimization algorithm

Our evolutionary algorithm consists in a Monte Carlo Simulated Annealing [40], which can be parallelized to evolve a population of sequences. Our approach consists in optimizing an objective function accounting for the interaction and structure of the RNAs that lead to the target behavior.

The design specifications comprise the secondary structures of all single RNAs, critical subsequences of nucleotides (e.g., RBS), the reaction free energies, and the structure of the output complex. The algorithm starts from pure random sequences satisfying the structural and subsequence constraints, although it can also be specified an initial sequence. If the subsequence constraints do not allow satisfying the structures, the algorithm stops. Eventually, we can introduce a relaxation in the structural constraints (through an harmonic constraint) allowing having species with dissimilar structures to their targets. Subsequently, an iterative process of mutation and selection is implemented (see scheme of the algorithm in Fig. S3). The mutation operator consists in either random or directed nucleotide replacements. We do not consider additions or deletions, so the length of the RNAs is maintained constant. To speed up the convergence, we generated a mutation operator that only created *useful* mutations, e.g., mutations that are always guaranteed to contribute for an interaction among RNA species. We do this by taking a word (i.e., set of consecutive nucleotides) from one sequence, making its reverse complementary, and randomly inserting it into another sequence. Initially, the length of this word is three, and it is reduced to one (i.e., single point mutation) during the optimization process. Those mutations speed up the *in silico* evolution. If a nucleotide that has to be mutated belongs to a stem, its pair in the stem is also mutated with the corresponding nucleotide with the aim of preventing the disruption of the secondary structure and improving the convergence. We avoid sequences having consecutive repeats of four or more identical nucleotides.

The objective function is a weighted sum of two terms to be minimized. The first term (Δ*G*_kin_) accounts for the reaction kinetics of the system. For that, we compute the Δ*G* and *α* of all possible reactions, having between species *A* and *B*

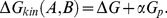
(13)Notice that Δ*G*_kin_ is a negative-valued variable. We will minimize or maximize Δ*G*_kin_ if the reaction must occur or not (in order to obtain the specified behavior). Maximizing Δ*G*_kin_ is equivalent to minimize −Δ*G*_kin_. During the optimization we exclude sequences forming homodimers. In addition, we considered Δ*G*_sat_ = −15 Kcal/mol and α_sat_ = 6 as arbitrary saturation levels (i.e., levels from which there is no need for further minimization). These values can be enlarged to get designs with lower Δ*G*_kin_, although at a cost of altering the convergence. The second term (Δ*G*_str_) accounts for the structural change of the output RNA. For that, we use a Hamming distance (*d*) between the current and target structures, being
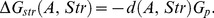
(14)This indicates that species *A* (which can be single or complex) is evolved to display the target structure, or substructure, *Str* (e.g., RBS paired, then repressing protein translation). *G_p_* is used to rescale the distance in terms of free energy. We note that Δ*G*_str_ is a positive-valued variable, which we will minimize.

### Experimental library of RNA devices

100 ng of plasmid pRAJ11 coding for the riboregulatory device RAJ11 were subjected to 30 cycles of PCR amplification with divergent primers I (5′-CCGCGAAGACCGGCACGGNNNGGTTGATTGTGTGAGTCTGTC-3′, N is A, C, G or T; BpiI recognition and cleavage sites underlined) and II (5′-GGCGGAAGACGCGTGCTCAGTATCTCTATCACTG-3′, BpiI recognition and cleavage sites underlined) in a volume of 20 µL with 0.4 U of the high fidelity Phusion DNA polymerase (Thermo Fisher Scientific) in the presence of HF buffer (Thermo Fisher Scientific), 3% dimethyl sulfoxide, 0.2 mM each dNTP and 0.5 µM each primer. Reactions consisted of an initial denaturation of 30 s at 98°C followed by 30 cycles of 10 s at 98°C, 30 s at 55°C and 1∶15 min at 72°C, with a final incubation of 10 min at 72°C. After PCR, 10 U of DpnI (Thermo Fisher Scientific) were added to each sample to digest the template plasmid and incubated for 1 h at 37°C. Reaction products were electrophoresed in a 1% agarose gel in TAE buffer (40 mM Tris, 20 mM sodium acetate, 1 mM EDTA, pH 7.2) and the gel stained with ethidium bromide. The 4460-bp long DNA product corresponding to the full-length plasmid was eluted from the gel, digested with BpiI for 1 h at 37°C (Thermo Fisher Scientific) and finally subjected to self-circularization with 5 U of T4 DNA ligase (Thermo Fisher Scientific) for 1 h at 22°C. Reaction products were purified by chromatography with silica gel spin columns (DNA Clean and Concentrator, Zymo Research) and electroporated in *E. coli* DH5α. Recombinant bacteria were selected in plates with 50 µg/mL ampicillin. Plasmids were purified from liquid cultures of selected clones (Wizard Plus SV Miniprep DNA Purification System, Promega) and analyzed by electrophoresis in 1% agarose gels in TAE buffer, followed by ethidium bromide staining. Forty-five plasmids whose electrophoretic mobility matched that of parental pRAJ11 were subjected to sequence analysis with primer III (5′-GAATTCGCGGCCGCTTCTAGAGC-3′) to find out the particular sequence in the randomized trinucleotide position introduced by primer I. Eleven mutant clones (see Table S3) were selected for further analysis, as well as the wild-type sRNA RAJ11 and the null system RAJ11m (Fig. S5).

### Characterization of RNA devices by fluorometry

Cultures (2 mL) inoculated from single colonies (three biological replicates) were grown overnight in LB medium at 37°C and 220 rpm. Cultures were then diluted 1∶100 (in 2 mL of LB), and were grown for 3 h in the same conditions (to reach an OD_600_ about 0.5). Ampicillin was used as antibiotic at 50 µg/mL. Then, 500 µL of each culture were centrifuged for 2 min at 13,000 rpm, and resuspended in the same volume of water. Subsequently, we loaded the multiwell plate with 200 µL for each sample, which was assayed in a Victor X5 (Perkin Elmer) to measure absorbance (600 nm absorbance filter) and fluorescence (485/14 nm excitation filter, 535/25 nm emission filter, for GFP). Background values of absorbance and fluorescence, which corresponded to water, were subtracted to correct the signals, and the normalized fluorescence was calculated as the ratio of fluorescence and absorbance (Fig. S4). Hence, we calculated the fold changes of activation (relative changes in GFP protein expression in absence or presence of sRNA).

## Supporting Information

Figure S1 **RNA secondary structures imposed for the different species in the designs.** The final structures may vary up to three base pairs.
(TIFF)

Figure S2 **Regulation of a natural gene.** Design of a synthetic sRNA (an analog of DsrA) able to interact with and release the RBS of the natural RpoS 5′ UTR. (A) Detail of the RpoS 5′ UTR, showing the RBS in blue and the start codon in green, together with the synthetic sRNA. (B) Detail of the intermolecular species.
(TIFF)

Figure S3 **Scheme of the algorithm to design riboregulation.**
(TIFF)

Figure S4 **Characterization results of our library of devices.** We present the fluorescence values for cells transformed with different plasmids: pRAJ11 and its derived mutants (mX), pRAJ11m, and pBS (pBlueScript, Stratagene) as a control. Error bars represent SE (standard errors).
(TIFF)

Figure S5 **Plasmid maps.** They correspond to the native RAJ11 device, which was previously engineered (Addgene refs. 39244 and 39245) [11].
(TIFF)

Table S1 **RNA sequences for the designs shown in the Figures.** On the 5′ UTRs, we highlight the RBS sequence (blue) and the start codon (red), and the poly(U) tail (yellow) when appropriate.
(DOC)

Table S2 **Properties of experimental systems for independent validation.** These RNA systems (selected from ref. [4] to cover a wide range of repression folds) are employed to validate the objective function used in this work. The regulatory data correspond to mutants of the natural system IS10. The systems were also expressed from plasmids in *E. coli*. Reported repression folds (changes in percentage of protein expression in absence or presence of sRNA) were measured by fluorometry.
(DOC)

Table S3 **RNA sequences of the library of devices constructed in this work.** These are mutants of the system RAJ11 (from ref. [11]). On the 5′ UTR, we highlight the RBS sequence (blue) and the start codon (red). Mutations on the sRNA highlighted in yellow.
(DOC)

Table S4 **Properties of our library of devices.** These RNA systems are employed to validate the objective function used in this work.
(DOC)
