# Supplementary figures and images for: Full Design Automation of Multi-State RNA Devices to Program Gene Expression Using Energy-Based Optimization
Source: PLoS Comput Biol. 2013 Aug 1;9(8):e1003172. doi: 10.1371/journal.pcbi.1003172 (PMC3731219; doi:10.1371/journal.pcbi.1003172)

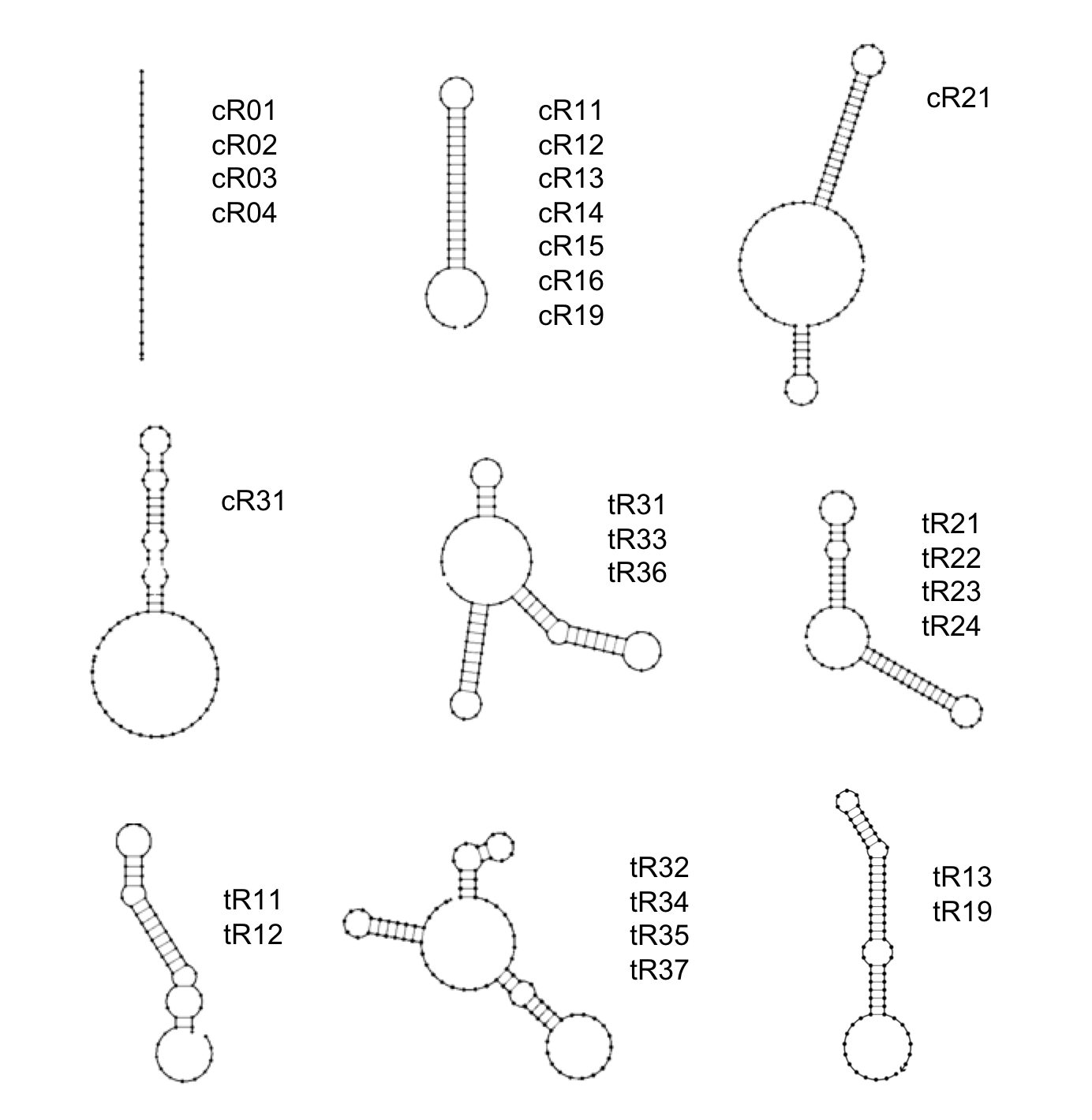

Supplement: Figure S1 — RNA secondary structures imposed for the different species in the designs. The final structures may vary up to three base pairs. (TIFF) [file pcbi.1003172.s001.tiff]

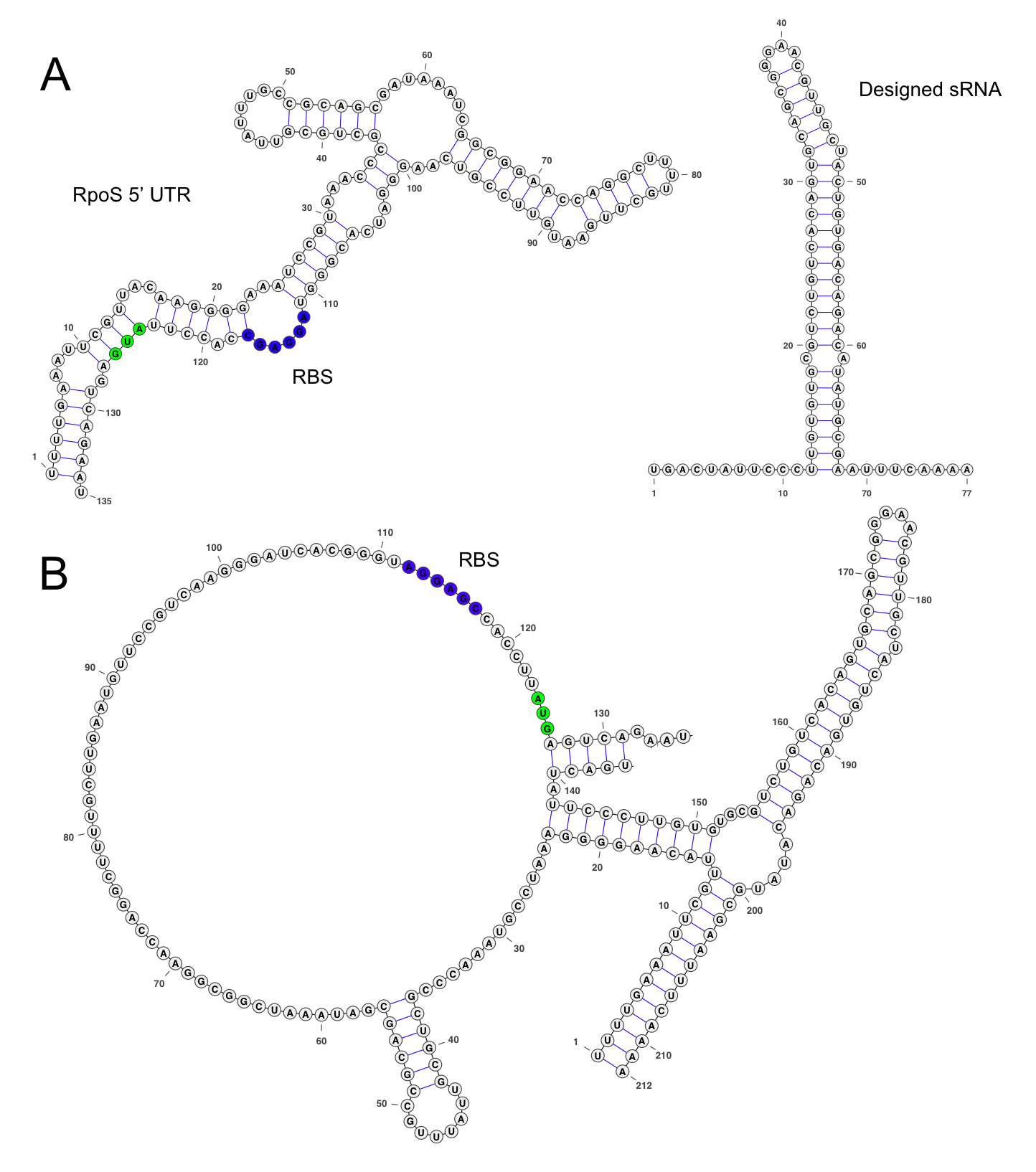

Supplement: Figure S2 — Regulation of a natural gene. Design of a synthetic sRNA (an analog of DsrA) able to interact with and release the RBS of the natural RpoS 5′ UTR. (A) Detail of the RpoS 5′ UTR, showing the RBS in blue and the start codon in green, together with the synthetic sRNA. (B) Detail of the intermolecular species. (TIFF) [file pcbi.1003172.s002.tiff]

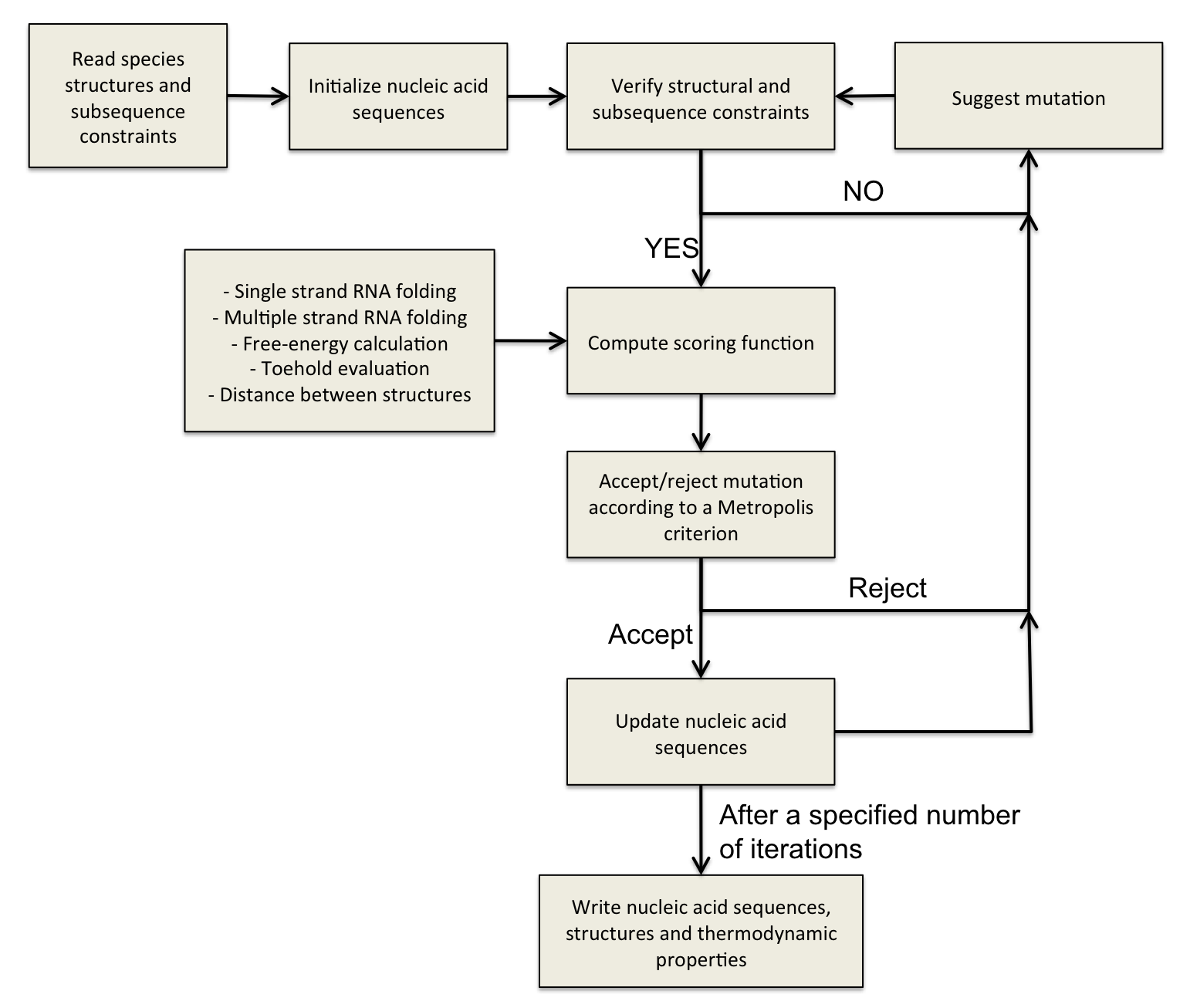

Supplement: Figure S3 — Scheme of the algorithm to design riboregulation. (TIFF) [file pcbi.1003172.s003.tiff]

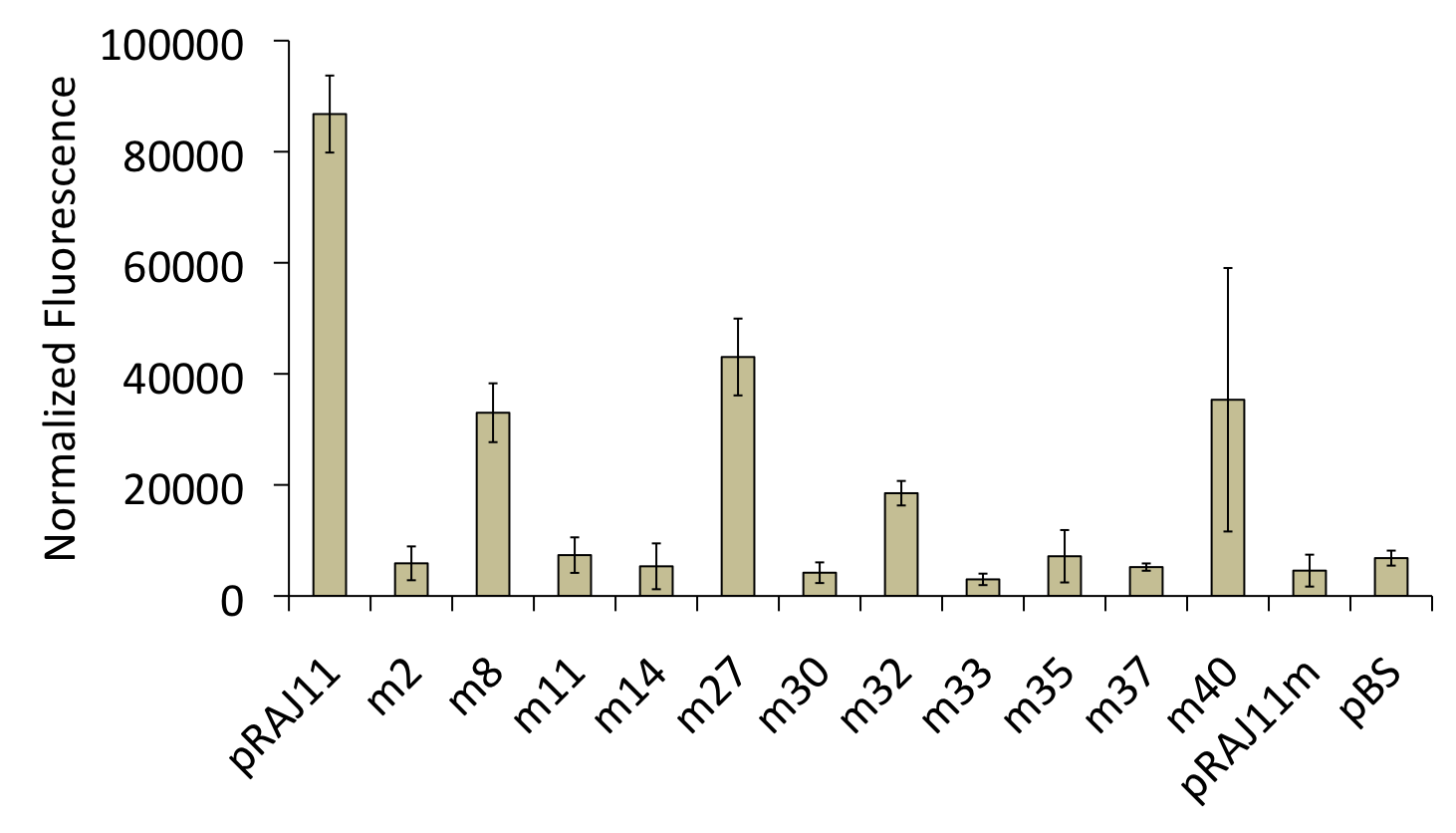

Supplement: Figure S4 — Characterization results of our library of devices. We present the fluorescence values for cells transformed with different plasmids: pRAJ11 and its derived mutants (mX), pRAJ11m, and pBS (pBlueScript, Stratagene) as a control. Error bars represent SE (standard errors). (TIFF) [file pcbi.1003172.s004.tiff]

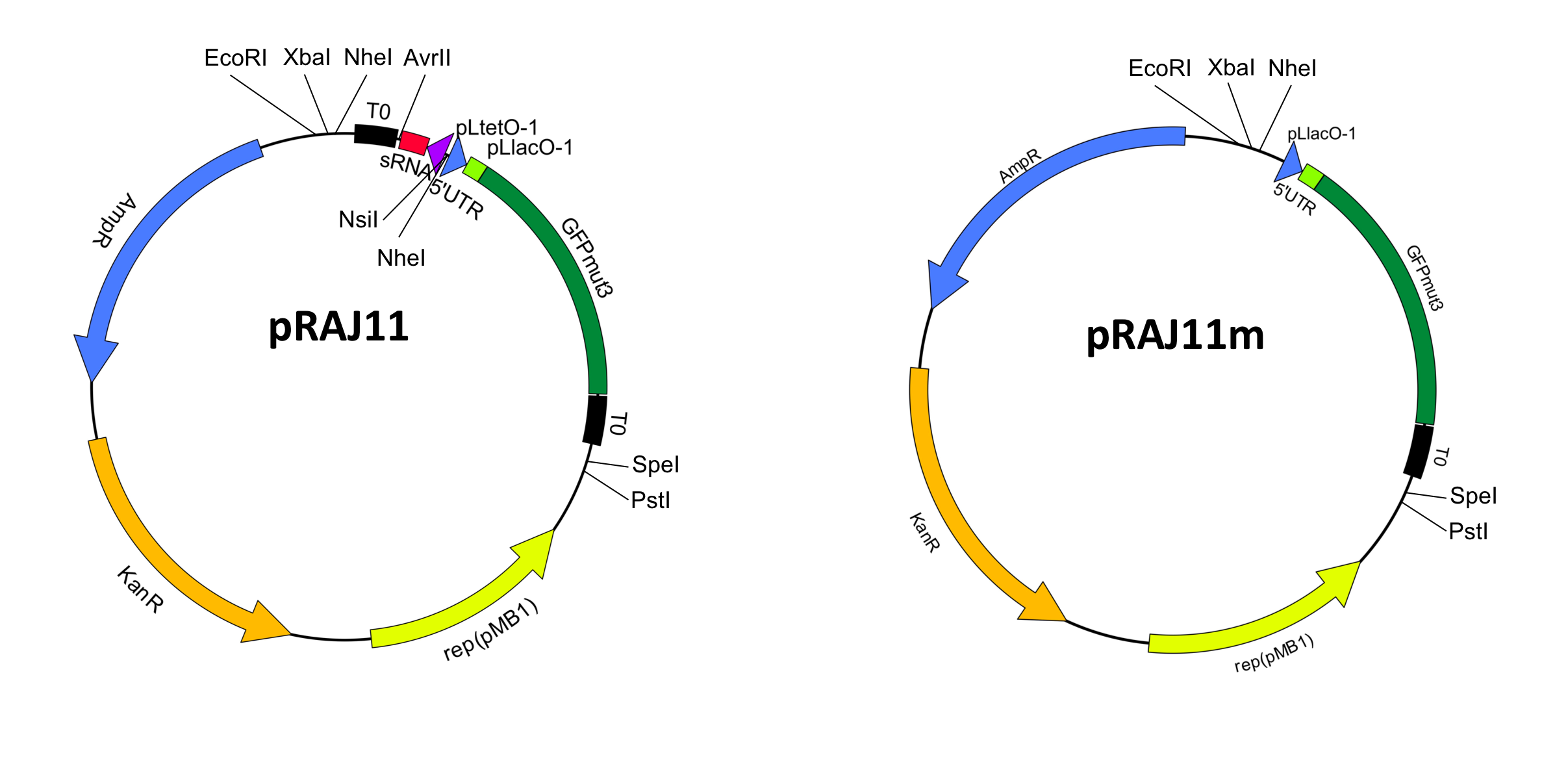

Supplement: Figure S5 — Plasmid maps. They correspond to the native RAJ11 device, which was previously engineered (Addgene refs. 39244 and 39245) [11]. (TIFF) [file pcbi.1003172.s005.tiff]
