# Supplementary material for: Full Design Automation of Multi-State RNA Devices to Program Gene Expression Using Energy-Based Optimization
Source: PLoS Comput Biol. 2013 Aug 1;9(8):e1003172. doi: 10.1371/journal.pcbi.1003172 (PMC3731219; doi:10.1371/journal.pcbi.1003172)
Supplement: Table S1 — RNA sequences for the designs shown in the Figures. On the 5′ UTRs, we highlight the RBS sequence (blue) and the start codon (red), and the poly(U) tail (yellow) when appropriate. (DOC) [file pcbi.1003172.s006.doc]

Table S1:

| **RNA id** | **RNA sequence** |
| --- | --- |
| **Fig. 3** |  |
| *cR01* | CCAACCAAAGAGGAGACACGACCAAUG |
| *tR31* | UUUCAAGGUGUCUCCCAUCUUGUGGUUGGUACAGGAGCCUUACCGGCUCCUGAUGCUAAUGGCGCGCUUGUAUUCCAGGCGCGCUAG |
| *cR02* | CAAGGGAAAGAGGAGACGAGGAGGAUG |
| *tR21* | UAUUCCUCGUUUCAGUCUUUCCACUGCGAUGAGGUCGUCAAGCCGAUUAUCUGUUUCUCGGAUAGUUGGUUUGUGUAGG |
| *cR03* | ACAGGUAAAGAGGAGACACGGGAUAUG |
| *tR11* | UCUUCUCUUUACCAGAGAUGUUACACGGCCAUAAUCGGCUUGUGACGUCAUCAGG |
| *cR04* | GAGAGUAAAGAGGAGAAUGAGAGGAUG |
| *tR32* | GCAUUCUCGUCUUUCGGUGCACAACGCCCUAGAGAUUUAUACCUACACCAGAUCUCCAGGUUUCCUCUACUACUGUAAUUCAGUAGUCAU |
| **Fig. 4** |  |
| *cR11* | GUGUCGGUUGUAUUUCUUCUUUAGGCACCCUCUUAAAGAGGAGAUGUAAUGUAUG |
| *tR33* | GGAGGGUCGCCUAAAGCGAUUUGUUUGCUCCCGCGUUCAUACCUAGGAUGUGUGGAGCAGAUCGGUCAUAAGGGAUAUGAUCGAUAU |
| *cR12* | UCCCAGGUGUUGUUUCCUCUUUGGUCGGGAAAUCAAAGAGGAGAUAACAUACAUG |
| *tR22* | CCGACCGAGAGAUUCAACACCUGGGACCUUUCGGAGCGCGGCUAUCUUCGGGUUGUGUUCUCGGAGGUAGUCGAACUCC |
| *cR13* | CCCUGCCUAGUAUCUCUUCUUUGCUUCCUCCAGUAAAGAGGAGAUAUUGGUUAUG |
| *tR12* | UGGAGGAGAGAGCGAUCCUAGUUCUCACUCAAAGAAGGGUAGGACUAGGCAGGGC |
| *cR14* | UCGGUCGUUAUAUUUCUUCUUUCAGGGUGGGCUGAAAGAGGAGAUAUGGCUAAUG |
| *tR34* | CGCUCACUGACACCCAAGCGACCAACAGUAGCGUAGCCCACCCUGAACAUACUGCGAUGCGAAGAAAUGGAAUAACGACCUUAUUUCUCG |
| **Fig. 5** |  |
| *cR21* | AUUUAUAACCACGAGAAUAAGGCUCGUGGACGGCUGCUUUCAACUUCAACUAGCGCCCUCGAUCUAGGGUGGGGAUCGAGGGUGCUGGUUUUUUUU |
| *tR35* | UGCCUCAGGACUAUCCGGCAGUAAUGAGGGAUCAGUCACCGACCGCCCUAGAUUGAGCCCGCUAGUUAUUCUCUUGACCCAAGGGAGCCU |
| *cR31* | GAAGCCACGCCAUACAGAGACCUUGCCUUUCUAAUGUCUCUUUGUAGCGUAAUUUACAAAGAGGAGAAUUAUACAAUG |
| *tR13* | GGGAGGGUUGAUUGUGUGAGUCUGUCACAGUUCAGCGGAAACGUUGAUGCUGUGACAGAUUUAUGCGAGGC |
| **Fig. 6** |  |
| *cR19* | CCUCGCAUAAUUUCACUUCUUCAAUCCUCCCGUUAAAGAGGAGAAAUUAUGAAUG |
| *tR19* | CAGAGGGAGGAUUGGAGGAGCGUGACUAGCGAGCUAUGGAAAUAGUUACGUUAGUCGCGGAUUUCUAGAGG |
| **Fig. 7** |  |
| *cR15* | AGUUCCGACGGGUCUCCUCUUUCGACUCCGCUUGAAAGAGGAGAUUUGUCAUAUG |
| *tR36* | CAAGUCCGUGAAGUGUACGGGCAGCUUGAUAUUUCGACCCUACCAGUUGGAACUAUUAAUUUGGGACCAUUCAUAGUGGUUCCGAAG |
| *tR23* | AAUUUAGGCGGAGUUGGGUAGAGGACGCUGCUUGUACGCUCUCGUAUUGACGGCACCCGCGUCGAUGUGAGGGACUUGG |
| *cR16* | AACAUGGAGCUAUUUCUUCUUUGGCCCUCUCGCUAAAGAGGAGAUAGCUUAAAUG |
| *tR24* | CUAACGCGAGAGGGUCUAUCAUCAUUGUCUCGCGCCUGGAUGACCUAUGCACUUACCAUGUGUAUAGGUUAUCAGCCCA |
| *tR37* | ACCUUGGGCGAAUAGCAGGUAUGAUACAGUGAUGAAAUGAGAAAGGAUAGCUUCAUGACUAAUUUAACAUCUAGUAGUGAAUUGGAUAAC |
