# Supplementary material for: Full Design Automation of Multi-State RNA Devices to Program Gene Expression Using Energy-Based Optimization
Source: PLoS Comput Biol. 2013 Aug 1;9(8):e1003172. doi: 10.1371/journal.pcbi.1003172 (PMC3731219; doi:10.1371/journal.pcbi.1003172)
Supplement: Table S2 — Properties of experimental systems for independent validation. These RNA systems (selected from ref. [4] to cover a wide range of repression folds) are employed to validate the objective function used in this work. The regulatory data correspond to mutants of the natural system IS10. The systems were also expressed from plasmids in E. coli. Reported repression folds (changes in percentage of protein expression in absence or presence of sRNA) were measured by fluorometry. (DOC) [file pcbi.1003172.s007.doc]

Table S2:

| 5’ UTR | sRNA | Repression fold (%) | -ΔG_kin_ (Kcal/mol) |
| --- | --- | --- | --- |
| S01 | A01 | 90 | 25.1 |
| S32 | A32 | 80 | 26.3 |
| S32 | A01 | 4 | 14.5 |
| S32 | A06 | 15 | 18.0 |
| S05 | A01 | 58 | 19.4 |
| S32 | A27 | 46 | 19.9 |
| S05 | A27 | 17 | 19.8 |
| S07 | A01 | 25 | 20.7 |
| S23 | A01 | 5 | 15.1 |
| S31 | A32 | 72 | 26.0 |
| S27 | A27 | 82 | 26.4 |
| S01 | A06 | 70 | 22.0 |
