# Supplementary material for: Full Design Automation of Multi-State RNA Devices to Program Gene Expression Using Energy-Based Optimization
Source: PLoS Comput Biol. 2013 Aug 1;9(8):e1003172. doi: 10.1371/journal.pcbi.1003172 (PMC3731219; doi:10.1371/journal.pcbi.1003172)
Supplement: Table S3 — RNA sequences of the library of devices constructed in this work. These are mutants of the system RAJ11 (from ref. [11]). On the 5′ UTR, we highlight the RBS sequence (blue) and the start codon (red). Mutations on the sRNA highlighted in yellow. (DOC) [file pcbi.1003172.s008.doc]

Table S3:

| **RNA id** | **RNA sequence** |
| --- | --- |
| *5’ UTR* | CCUCGCAUAAUUUCACUUCUUCAAUCCUCCCGUUAAAGAGGAGAAAUUAUGAAUG |
| *RAJ11* | GGGAGGGUUGAUUGUGUGAGUCUGUCACAGUUCAGCGGAAACGUUGAUGCUGUGACAGAUUUAUGCGAGGC |
| *RAJ11-m2* | GGCCCGGUUGAUUGUGUGAGUCUGUCACAGUUCAGCGGAAACGUUGAUGCUGUGACAGAUUUAUGCGAGGC |
| *RAJ11-m8* | GGGGGGGUUG_UUGUGUGAGUCUGUCACAGUUCAGCGGAAACGUUGAUGCUGUGACAGAUUUAUGCGAGGC |
| *RAJ11-m11* | GGGUUGGUUGAUUGUGUGAGUCUGUCACAGUUCAGCGGAAACGUUGAUGCUGUGACAGAUUUAUGCGAGGC |
| *RAJ11-m14* | GGUUUGGUUGAUUGUGUGAGUCUGUCACAGUUCAGCGGAAACGUUGAUGCUGUGACAGAUUUAUGCGAGGC |
| *RAJ11-m27* | GGGGGGGUUGAUUGUGUGAGUCUGUCACAGUUCAGCGGAAACGUUGAUGCUGUGACAGAUUUAUGCGAGGC |
| *RAJ11-m30* | GGUAGGGUUGAUUGUGUGAGUCUGUCACAGUUCAGCGGAAACGUUGAUGCUGUGACAGAUUUAUGCGAGGC |
| *RAJ11-m32* | GGGUGGGUUGAUUGUGUGAGUCUGUCACAGUUCAGCGGAAACGUUGAUGCUGUGACAGAUUUAUGCGAGGC |
| *RAJ11-m33* | GGAAAGGUUGAUUGUGUGAGUCUGUCACAGUUCAGCGGAAACGUUGAUGCUGUGACAGAUUUAUGCGAGGC |
| *RAJ11-m35* | GGUCGGGUUGAUUGUGUGAGUCUGUCACAGUUCAGCGGAAACGUUGAUGCUGUGACAGAUUUAUGCGAGGC |
| *RAJ11-m37* | GCAGGGGUUGAUUGUGUGAGUCUGUCACAGUUCAGCGGAAACGUUGAUGCUGUGACAGAUUUAUGCGAGGC |
| *RAJ11-m40* | GGCGGGGUUGAUUGUGUGAGUCUGUCACAGUUCAGCGGAAACGUUGAUGCUGUGACAGAUUUAUGCGAGGC |
