# Supplementary material for: Full Design Automation of Multi-State RNA Devices to Program Gene Expression Using Energy-Based Optimization
Source: PLoS Comput Biol. 2013 Aug 1;9(8):e1003172. doi: 10.1371/journal.pcbi.1003172 (PMC3731219; doi:10.1371/journal.pcbi.1003172)
Supplement: Table S4 — Properties of our library of devices. These RNA systems are employed to validate the objective function used in this work. (DOC) [file pcbi.1003172.s009.doc]

Table S4:

| RNA system | Fold change of activation | -ΔG_kin_ (Kcal/mol) |
| --- | --- | --- |
| RAJ11 | 19.00 | 24.4 |
| RAJ11-m2 | 1.28 | 8.8 |
| RAJ11-m8 | 7.22 | 23.0 |
| RAJ11-m11 | 1.61 | 12.4 |
| RAJ11-m14 | 1.17 | 8.5 |
| RAJ11-m27 | 9.42 | 23.6 |
| RAJ11-m30 | 0.92 ^*^ | 13.8 |
| RAJ11-m32 | 4.05 | 16.4 |
| RAJ11-m33 | 0.66 ^*^ | 8.7 |
| RAJ11-m35 | 1.56 | 11.4 |
| RAJ11-m37 | 1.14 | 12.9 |
| RAJ11-m40 | 7.73 | 13.8 |

^*^ These values were set to 1 to plot Fig. 2A.
